# The Fusion Activity of IM30 Rings Involves Controlled Unmasking of the Fusogenic Core

**DOI:** 10.3389/fpls.2019.00108

**Published:** 2019-02-07

**Authors:** Adrien Thurotte, Dirk Schneider

**Affiliations:** Institute of Pharmacy and Biochemistry, Johannes Gutenberg University, Mainz, Germany

**Keywords:** chloroplast, membrane biogenesis, membrane fusion, thylakoid membrane, cyanobacteria, Vipp1, IM30, PspA

## Abstract

The *inner membrane-associated protein of 30 kDa* (IM30, also known as Vipp1) is required for thylakoid membrane biogenesis and maintenance in cyanobacteria and chloroplasts. The protein forms large rings of ∼2 MDa and triggers membrane fusion in presence of Mg^2+^. Based on the here presented observations, IM30 rings are built from dimers of dimers, and formation of these tetrameric building blocks is driven by interactions of the central coiled-coil, formed by helices 2 and 3, and stabilized via additional interactions mainly involving helix 1. Furthermore, helix 1 as well as C-terminal regions of IM30 together negatively regulate ring-ring contacts. We propose that IM30 rings represent the inactive form of IM30, and upon binding to negatively charged membrane surfaces, the here identified fusogenic core of IM30 rings eventually interacts with the lipid bilayer, resulting in membrane destabilization and membrane fusion. Unmasking of the IM30 fusogenic core likely is controlled by Mg^2+^, which triggers rearrangement of the IM30 ring structure.

## Introduction

The development of oxygenic photosynthesis in algae and plants via the endosymbiotic acquisition of an ancestor of modern days’ cyanobacteria was key for the evolution of aerobic life on earth (Zimorski et al., 2014). Due to their relatedness, the ultrastructure of cyanobacteria and chloroplasts is preserved, and in both the photosynthetic light reaction is typically localized in a unique cellular compartment, the thylakoid membrane (TM) system. Although, many processes occurring at/in TMs have been analyzed to a great extent and are well described, the biogenesis and dynamics of the TM itself are yet to be fully understood (Nickelsen and Zerges, 2013; Rast et al., 2015). In fact, in both cyanobacteria and chloroplast, the structure of the TM network is highly dynamic and fusion of different TM stacks have already been observed in cyanobacteria as well as in chloroplasts of green algae and higher plants (Shimoni et al., 2005; van de Meene et al., 2006; Nevo et al., 2007; Chuartzman et al., 2008; Liberton et al., 2011a,b; van de Meene et al., 2012; Engel et al., 2015). Such dynamic (re)organization of the TM network appears to be critical for optimizing the photosynthetic light reactions (Chuartzman et al., 2008; Barthel et al., 2012; Charuvi et al., 2012; Nevo et al., 2012; Pribil et al., 2014; Flori et al., 2017). Transient connections between the cyanobacteria cytoplasmic membrane (CM) and TMs have been observed (van de Meene et al., 2012), as well as fusion of the chloroplast inner envelope membrane (IE) with TMs (Shimoni et al., 2005; Charuvi et al., 2012; Engel et al., 2015). However, these observations raise the question as to the molecular mechanisms triggering such membrane fusion and fission events.

The *inner membrane-associated protein of 30 kDa* (IM30, also named Vipp1: the *vesicle-inducing protein in plastids 1*) has been observed to be able to mediate membrane fusion, at least *in vitro* (Hennig et al., 2015). IM30 is present in chloroplasts and in TM-containing organisms but is absent in the TM-free cyanobacterium *Gloeobacter violaceus* (Nakamura et al., 2003; Vothknecht et al., 2012). In plants, depletion of IM30 disturbs the TM ultrastructure (Kroll et al., 2001; Nordhues et al., 2012), and defects in osmotic pressure regulation (Zhang et al., 2012) as well as a high sensitivity to oxidative stress were observed (Zhang et al., 2012, 2016b). Besides an abnormal TM structure, impaired photosystem II (PS II) assembly has been observed in the green alga *Chlamydomonas reinhardtii* (Nordhues et al., 2012), whereas a decreased PS I content has been reported in the cyanobacteria *Synechocystis* sp. PCC 6803 (Fuhrmann et al., 2009b) and *Synechococcus* sp. PCC 7002 (Zhang et al., 2014) when IM30 is depleted or deleted.

Together with the phage shock protein A (PspA), IM30 is part of the PspA/IM30 protein family of membrane chaperones (Vothknecht et al., 2012; Thurotte et al., 2017). All members of the PspA/IM30 family appear to be able to bind to membrane surfaces, and a membrane protecting activity has been suggested (Kobayashi et al., 2007; Zhang and Sakamoto, 2013; Thurotte et al., 2017). While not essential, PspA proteins are encoded in several bacterial species, whereas expression of IM30 is vital in chloroplasts and cyanobacteria, albeit cyanobacteria also encode PspA (Vothknecht et al., 2012). Nevertheless, IM30 and PspA appear to have different physiological functions, at least in part, as e.g., expression of *pspA* does not complement *im30*-defective cyanobacteria (Westphal et al., 2001; Hennig et al., 2017). Thus far, a membrane fusion activity has been described for IM30 but not for PspA and thus, this activity likely differentiates IM30 from PspA (Junglas and Schneider, 2018).

Nevertheless, both, PspA and IM30 proteins, have similar structures and both form higher-ordered oligomeric rings (Hankamer et al., 2004; Fuhrmann et al., 2009a; Saur et al., 2017; Wolf, 2017), which are built from repeating building blocks, likely PspA/IM30 tetramers (Hankamer et al., 2004; Bultema et al., 2010; Vothknecht et al., 2012; Heidrich et al., 2016). However, PspA might also form hexameric (sub-) structures (Elderkin et al., 2005; Joly et al., 2009). While in case of PspA solely ring assemblies of 9 basal building blocks are described (Hankamer et al., 2004), IM30 displays a remarkable structural variability, and rings of 9–24 building blocks have been identified thus far (Fuhrmann et al., 2009a; Saur et al., 2017). However, neither the physiological role of this ring diversity nor the exact monomer or tetramer organization within these rings are determined and/or understood yet.

The secondary structures of PspA and IM30 monomers is highly conserved. Both proteins are largely α-helical and six consecutive α–helical regions, interrupted by short unstructured areas, are predicted for both proteins (Vothknecht et al., 2012). Based on the crystal structure of a PspA fragment (residues 1–144, comprising helices 1–3), the long helices 2 and 3 form an extended coiled-coil (Osadnik et al., 2015). In contrast to PspA, an extra C-terminal helix, helix 7, is predicted for IM30 proteins that is separated from helix 6 via an extended unstructured region (Zhang et al., 2016a; Hennig et al., 2017). This IM30-specific helix 7 extrudes from the ring, and it has been suggested that this helix is important for properly localizing IM30 at the inner membrane of *A. thaliana* chloroplasts (Aseeva et al., 2004; Zhang et al., 2016a) as well as at cyanobacterial membranes (Hennig et al., 2017). This C-terminal helix 7 associates with the lipid bilayer, is important for protecting chloroplast membranes against stress and can modulate membrane fusion (Zhang et al., 2016a; Hennig et al., 2017). The latter observation suggests that a precise organization of individual IM30 proteins and of helix 7 within the higher-ordered ring structures is crucial for the proteins’ fusion activity. However, the contribution of other protein regions and the minimal sequence allowing ring formation and membrane fusion remain controversial or essentially enigmatic thus far.

In the present work, we have assessed the role of individual IM30 α-helices for membrane fusion and ring formation using truncated IM30 proteins. Based on our observation, the coiled-coil formed by the extended helices 2 and 3 appears to be the fusogenic domain of IM30. This fusion core is shielded in the IM30 ring structures, which are formed and stabilized via interactions involving helices 1 and 4–6. Based on the observed structures, the membrane binding affinities and the membrane fusion activities of the analyzed proteins, we propose that IM30 rings represent the inactive form of IM30. Only upon binding to negatively charged membrane surfaces, the fusogenic core of IM30 rings eventually interacts with the lipid bilayer resulting in membrane destabilization and membrane fusion.

## Materials and Methods

### Cloning and Expression of IM30 Variants

Cloning, expression and purification of the *Synechocystis* IM30 wt protein are described in detail in Fuhrmann et al. (2009a). Plasmids for expression of truncated IM30 were created via PCR-based mutagenesis following the protocol described in Liu and Naismith (2008). In the case of C-terminal truncated proteins, artificial stop codons were introduced via the primers (Table 1). For expression of N-terminally truncated proteins, primers were designed to delete the corresponding 3′ gene region. The sequence of each construct was verified by DNA sequencing. Truncated proteins were expressed and purified as the wt.

**Table 1:** Primers used for construction of IM30 expression plasmids.

Primers for stop codon insertion	Sequence →(5′ 3′)
*α16-fw*	TATGTCATATGGGATTATTTGACCGTTTAGG
*α16-rev*	ATAGCGGATCCTTAGGAAGCTTTCAGGGCGGCC
*α14-fw*	GCAGGGGAGTTAGCCTGATTTGGCATCGAGAACC
*α14-rev*	GGTTCTCGATGCCAAATCAGGCTAACTCCCCTGC
*α13-fw*	GGCCAGGGCCAAATAGGCCAAGGCTAATGCTG
*α13-rev*	CAGCATTAGCCTTGGCCTATTTGGCCCTGGCC
**Primers for N-terminal deletions**	
*NT deletion rem-rev*	CATCATCACAGCAGCGGCCATATCGACGACGACGACAAGCAT
*α1 removal-fw*	AACTTTTTCTGGATCTTCAGCATGCTTGTCGTCGTCGTCGAT
*α13 removal-fw*	CTGCAGTTCAGCATTAGCCTTATGCTTGTCGTCGTCGTCGAT


### Circular Dichroism (CD) Spectroscopy

The secondary structure and stability of the proteins (0.1 mg/mL) was determined via CD spectroscopy. Spectra were recorded in 1 nm steps in 10 mM HEPES buffer (pH 7.6) at 25°C using a Jasco-815 CD spectrometer and a path length of 1 mm. Five spectra were averaged and smoothed using the Savitzky-Golay algorithm integrated in the JASCO software package. Three individual samples were measured and the spectra were converted to molar ellipticity. For thermal denaturation, three spectra were converted to molar ellipticity and averaged at each temperature. Spectra were measured every 2°C from 15°C to 95°C. The value at 222 nm was plotted against the temperature, and the resulting curve was fitted by a Boltzmann function to determine the melting temperature.

### Electron Microscopy and Image Processing

Negatively stained samples were prepared as described recently (Saur et al., 2012, 2017). In brief: the sample (5 uL of a 0.2 mg/mL protein solution) was pipetted onto a negatively glow discharged continuous carbon grid [30 s at 25 mA in an Emitech K100X glow discharge system (Quorum Technologies Ltd.)]. After one minute, the samples were washed with 3 × 20 μL of distilled H_2_O, blotted on the edge of the grid with a filter paper, contrasted for 45 s with 5 μl of 2% uranyl acetate staining solution, and then blotted dry on the edge of the grid with filter paper. The pictures were taken with a FEI Tecnai 12 electron microscope (acceleration voltage: 120 kV, CS = 6.3 mm; nominal magnification: 71, 540×; nominal under focus: 0.5–1.5 μm) on a TVIPS TemCam-F416 4 K CCD camera.

### Liposome Preparation

DOPG (1,2-dioleoyl-*sn*-glycero-3-phosphoglycerol), DOPC (1,2-dioleoyl-*sn*-glycero-3-phosphocholine), MGDG (monogal actosyldiacylglycerol) and DGDG (digalactosyldiacylglycerol), as well as the fluorescence dyes NBD-PE (1,2-distearoyl-*sn*-glycero-3-phosphoethanolamine-*N*-(7-nitro-2-1,3-benzoxadiazol-4-yl)) and LissRhodPE [Lissamin Rhodamin PE; 1,2-Dioleoylsn-glycero-3-phosphoethanolamin-*N*-(lissamin-rhodamin-B-sulfonyl)] were purchased from Avanti Polar Lipids, Inc. (Birmingham, AL, United States).

The organic solvent (chloroform/methanol 2:1 (v/v)) was removed under a gentle stream of nitrogen gas and overnight vacuum desiccation to allow the formation of a lipid film. Lipids were hydrated in 20 mM HEPES, pH 7.6 buffer and unilamellar liposomes were prepared by five cycles of freezing in liquid nitrogen and thawing at 37°C, followed by 15 extrusions through a 100 nm filter (Nucleopore Track-Etch Membrane, Whatman, Sigma-Aldrich, Taufkirchen, Germany), using an extruder (Avanti Polar Lipids, Inc., Birmingham, AL, United States).

### Laurdan Fluorescence Spectroscopy

Laurdan (Sigma, Taufkirchen, Germany) is a fluorescent dye that incorporates into the lipid bilayer and allows quantifying membrane lipid order (Parasassi et al., 1991; Parasassi and Gratton, 1995). The dye was added to 0.5 mM lipid solutions prior to liposome formation at a molar ratio of 1:500. After addition of the protein and incubation for 30 min at room temperature, Laurdan fluorescence emission was determined at 25°C upon excitation of the dye at 350 nm using a FluoroMax-4 fluorescence spectrometer from Horiba Scientific, Kyoto, Japan. The fluorescence emission spectrum of Laurdan depends on the physical state of the surrounding lipid bilayer. The Laurdan generalized Polarization (GP) value reflects the lipid order (Parasassi et al., 1991) and is calculated according to equation 1, where I_440_ and I_490_ are the fluorescence emission intensities at 440 and 490 nm, respectively.

(1) $$Laurdan\;GP=\frac{I_{440}-I_{490}}{I_{440}+I_{490}}$$

### Membrane Fusion Activity

IM30-induced membrane fusion was quantified using an assay based on Förster resonance energy transfer (FRET), as described in Hennig et al. (2015). Sized (100 nm) liposomes (MGDG/DOPG, 60/40) were labeled with two lipid-anchored dyes [0.8 mol% LissRhod-PE (FRET-donor) and NBD-PE (FRET-acceptor)] and mixed with unlabeled liposomes in large excess (1:9). When labeled liposomes fuse with unlabeled, the chromophores are separated in space, FRET is drastically decreased and thus, the fluorescence of the FRET donor dye increases. The fluorescence of the donor chromophore was monitored over time at 535 nm after excitation at 460 nm. The slit widths were set to 5 nm. Unless otherwise mentioned, the measurements were performed in presence of 2.5 μM protein, 0.1 mM lipids and 7.5 mM Mg^2+^ (Hennig et al., 2015). Three independent measurements were averaged for each IM30 variant and the SD is given.

### Size Exclusion Chromatography (SEC)

SEC analyses were performed using an Äkta Basic System (Amersham Biosciences, Freiburg, Germany) and a Superdex 200 HR 16/60 GL column (4°C, 0.5 mL/min flow rate, detection wavelength: 280 nm). The column was equilibrated with 20 mM HEPES buffer, pH 7.6. For calibration, the following molecular mass markers were analyzed: blue dextran (>2,000 kDa), ferritin (440 kDa), β-amylase (200 kDa), alcohol dehydrogenase (150 kDa), conalbumin (75 kDa), bovine serum albumin (66 kDa), ovalbumin (43 kDa), carbonic anhydrase (29 kDa) and ribonuclease A (14.7 kDa). The gel phase distribution coefficient K_av_ was used for estimation of molecular masses.

## Results

### Using Truncated IM30 to Identify IM30 Domains of Structural and/or Functional Importance

**FIGURE 1:**
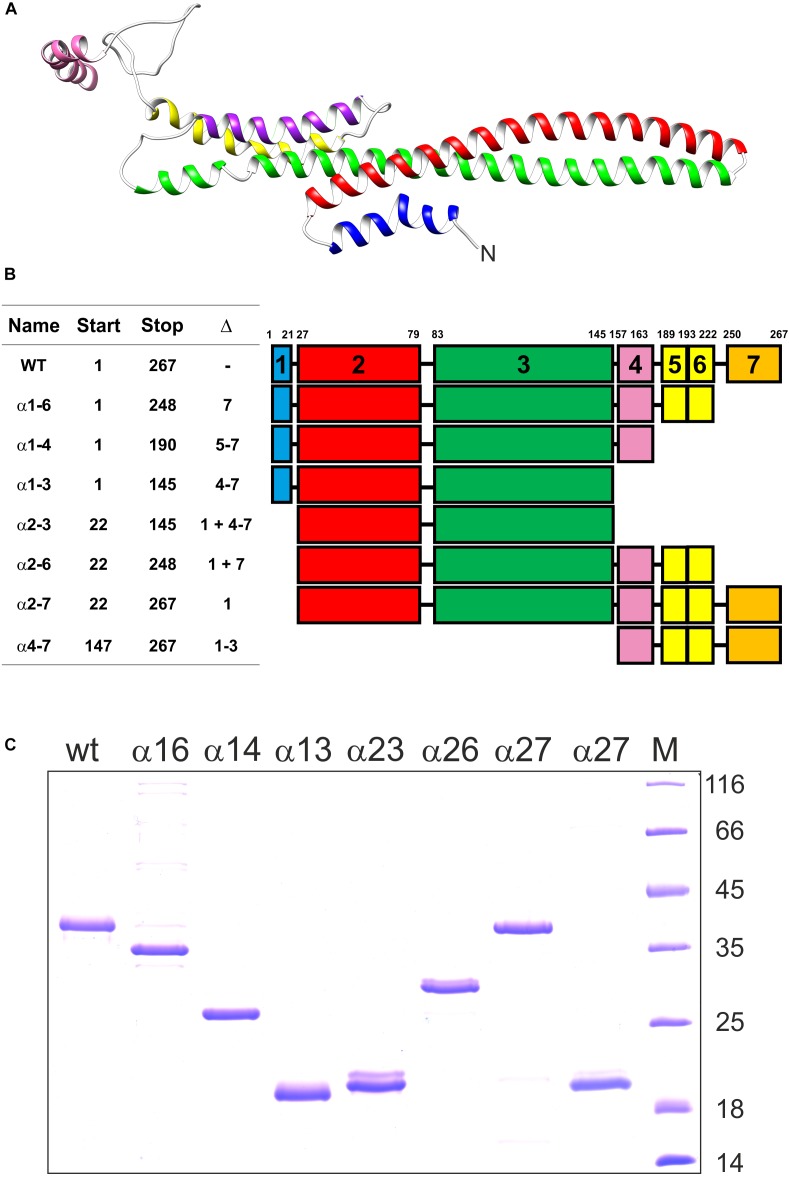
Truncated IM30 proteins analyzed in this study. **(A)** The predicted structure of an IM30 monomer according to (Saur et al., 2017). The N-terminus of the protein is indicated. **(B)** The IM30 mutants analyzed in this study. The names correspond to the α-helices present in the expressed and analyzed proteins. Start and Stop refers to the corresponding amino acids, and Δ to the α-helices removed. The region between amino acid 189-222 likely forms a single α-helix (Heidrich et al., 2016; Saur et al., 2017), and, consequently, this region was named *helix 5/6*. **(C)** SDS PAGE of the heterologously expressed and purified IM30 proteins analyzed in this study. M: molecular mass standard. The molecular masses of the reference proteins (in kDa) is given on the right hand side.

IM30 forms large fusogenic ring structures. Recent structural analyses have indicated that these rings assemble from tetrameric building blocks (Heidrich et al., 2016; Saur et al., 2017). Yet, which regions of the monomeric IM30 protein are crucial for oligomerization and/or are involved in membrane interaction and the IM30-inherent membrane fusion activity is still largely enigmatic. We have tackled this question via generating and analyzing truncated IM30 proteins. We anticipated that this would allow pinpointing protein regions crucial for assembly and/or activity of IM30 rings. Therefore, we have expressed and purified various shortened forms of IM30 of the cyanobacterium *Synechocystis* sp. PCC 6803, the thus far best characterized IM30 protein. Based on the structure of a PspA fragment (Osadnik et al., 2015), the coiled-coil formed by the helices 2 and 3 is the structural core of members of the PspA/IM30 protein family (Figure 1A). Thus, we have created, expressed, purified and analyzed the wt protein plus six different IM30 versions, which all contained the helix 2/3 core (Figure 1B) but were truncated at the N- (helix 1) and/or the C-terminus (helices 4–7). We have refrained from deleting the predicted helix 6 alone, as helix 5 and 6 is predicted to form a single, continuous α-helix (Figure 1A; Heidrich et al., 2017). In addition, we have also expressed and purified a truncated IM30 variant that contains solely the C-terminal 4–7 (amino acids 147–267).

Expression and purification of the proteins is described in detail in the M&M section. The purified proteins (Figure 1C) were subsequently analyzed *in vitro* to identify domains crucial for the structure and activity of IM30.

### Helix 5/6 Is Needed for IM30 Ring Formation

**FIGURE 2:**
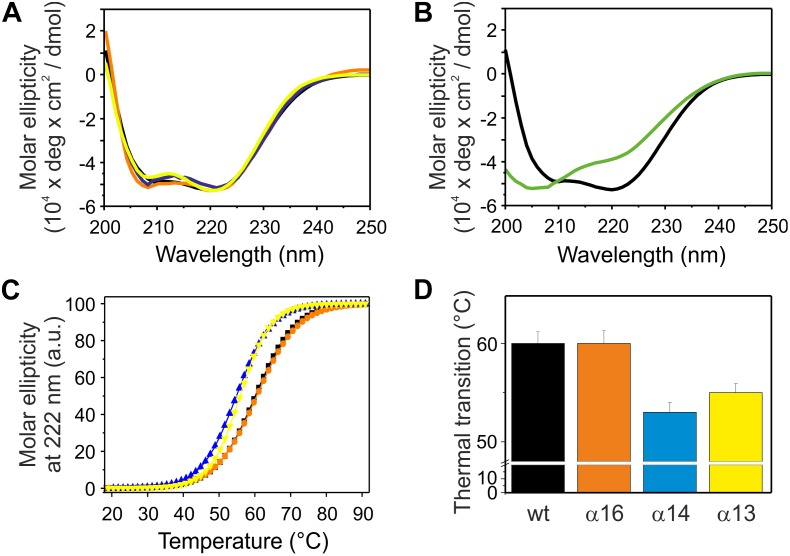
Structure and stability of C-terminally truncated IM30. **(A)** CD spectra of wt (black), α16 (orange), α14 (blue), and α13 (yellow) IM30. **(B)** CD spectra of wt (black) and α4-7 (green). **(C)** The CD signal at 222 nm of wt (black), α16 (orange), α14 (blue), and α13 (yellow) IM30 was followed at increasing temperatures, and the data (melting curves) were fitted with a Boltzmann function (*R*^2^ > 0.95) yielding the transition point (i.e., the melting point) **(D)**. Error bars represent SD of three independent measurements.

Besides the coiled-coil core helices 2 and 3, three additional α-helical domains are predicted within the IM30 C-terminal region (helices 4, 5/6 and 7; Figure 1A,B). As can be seen in Figure 2, the CD spectra of all isolated C-terminally shortened fragments are characteristic for largely α-helical proteins and show minima at 208 nm and 222 nm, as expected based on the proposed IM30 structure (Figure 1A; Saur et al., 2017). In contrast, the CD-spectrum of the C-terminal region, corresponding to helices 4–7, did not indicate formation of extended α-helical regions (Figure 2B). Rather, this protein fragment was essentially unstructured in solution. Thus, we refrained from further analyzing this protein fragment.

Next, the stability of the C-terminally truncated proteins was determined via following temperature-induced changes of the CD signal at 222 nm (Figure 2C). For each construct the melting temperature was determined from the inflection point of the melting curves gained via fitting of the data (Figure 2D). Two of the analyzed C-terminally truncated proteins have a substantially lowered thermal stability, and solely IM30 α1-6 was as stable as the wt protein, in line with recent observations (Hennig et al., 2016). The melting temperature of α1-3 is lowered by about 5°C and did not increase when helix 4 was present (α1-4). Thus, deletion of the C-terminally located α-helices 5/6 significantly decreases the stability of the IM30 secondary structure.

IM30 monomers have been shown to assemble into stable tetrameric sub-structures that successively form large oligomeric ring structures (Heidrich et al., 2016; Saur et al., 2017). To identify IM30 helices crucial for quaternary structure formation, we next analyzed the C-terminally truncated proteins via size exclusion chromatography (SEC), which allows separation of different oligomeric IM30 species.

**FIGURE 3:**
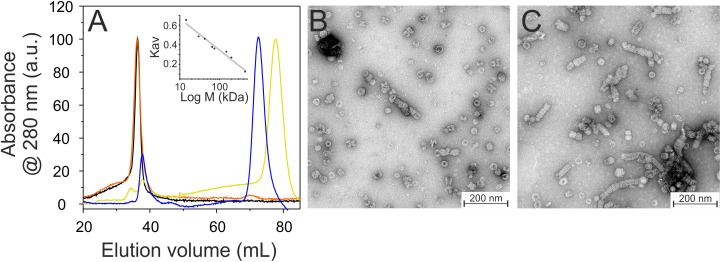
Oligomeric structure of wt (black), α16 (orange), α14 (blue), and α13 (yellow) IM30. **(A)** Size exclusion profiles of C-terminally truncated IM30 versions. The wt and α16 proteins mainly eluted in the void volume as high molecular mass oligomers, whereas α14 and α13 eluted as smaller oligomers. The apparent molecular mass of the fractions has been determined using a calibration curve (inlet) using the proteins described in the Methods section. **(B)** Negatively stained transmission electron micrographs of wt (B) and α16 **(C)** proteins.

Based on our SEC analyses, solely the wt protein and the C-terminally truncated protein α1-6 form high molecular mass oligomers (Figure 3A). Further C-terminal truncation, i.e., removal of at least helix 5/6, abolished formation of such high molecular mass oligomers and resulted in formation of smaller oligomeric structures. The α1-3 and α1-4 proteins eluted at around 78.6 mL and 73.3 mL, respectively (Figure 3A). With calculated molecular masses of ∼33.6 and 52.0 kDa, these values suggest formation of dimeric structures (Table 2), as further discussed below.

**Table 2:** SEC analyses of truncated IM30.

Protein	Elution peak (mL)	K_av_	MM extrapolated	Calculated MM of monomer in kDa	Apparent oligomeric state
α1-4	73.3 ± 1.1	0.43 ± 0.012	52.0 ± 4.5	23.7	2.2 ± 0.20
α1-3	78.6 ± 1.2	0.49 ± 0.014	33.6 ± 3.3	19.1	1.76 ± 0.19
α2-3	85.3 ± 0.4	0.56 ± 0.005	19.3 ± 0.7	16.8	1.15 ± 0.042
	90.0 ± 1.8	0.62 ± 0.021	13.1 ± 2.0		0.78 ± 0.14


*Elution peak maxima (gained from the SEC profiles shown in Figure 3, 6, 7) and the calculated gel phase distribution coefficient (K_av_) were used to extrapolate apparent molecular masses (MM) in kDa. The extrapolated MMs were divided by the calculated MMs (including the His-tag) of the respective constructs (monomer) to estimate the oligomeric state. The colors refer to the respective SEC profiles shown in Figure 3A, 7D.*

While formation of high molecular mass oligomeric structures is indicated by the SEC analyses for the wt and α1-6 proteins, the results did not reveal whether the truncated IM30 protein still forms the typical IM30 ring structure or unspecific aggregates, as IM30 rings are not explicitly separated and elute in the void volume. Thus, we next analyzed ring formation via transmission electron microscopy (TEM). As can be seen in Figure 3B,C, the IM30 wt and the α1-6 proteins both form prototypical IM30 rings, whereas the remaining truncated proteins did not form higher ordered structures (data not shown), in line with the SEC analyses. Furthermore, in case of the α1-6 protein, an increased tendency to form rod structures via ring stacking was observed (Figure 3C).

**FIGURE 4:**
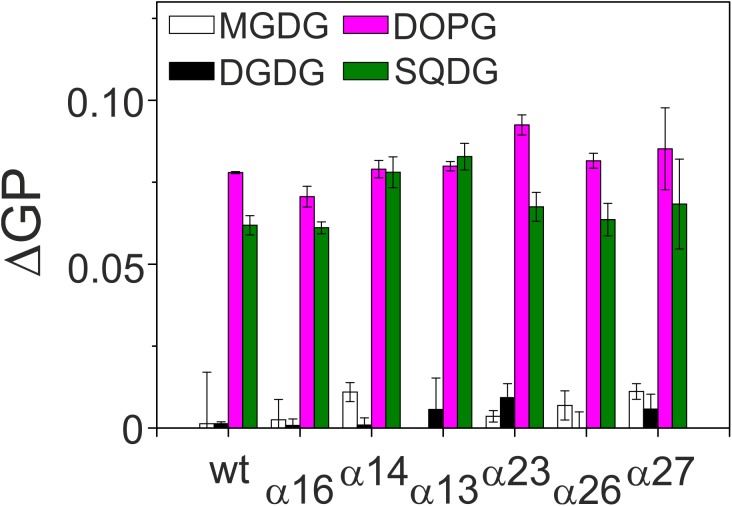
Membrane binding affinity of IM30 proteins. Peripheral binding of IM30 to TM lipid-containing liposomes was followed by monitoring changes in the Laurdan ΔGP value. Increased ΔGP values indicate stronger binding of the protein to the respective membrane surface. Each lipid composition was analyzed at least three times and the SD is given.

IM30 rings have been shown to interact in a well-defined geometry, specifically with negatively charged membrane surfaces (Hennig et al., 2015). Nevertheless, the membrane interacting regions are not well defined yet, albeit recent results suggest that the terminal helices 1 and 7, as well as the loop between helices 2 and 3 could be involved in membrane binding (Jovanovic et al., 2014b; Hennig et al., 2015; McDonald et al., 2017; Saur et al., 2017). To identify (additional) IM30 regions involved in membrane attachment, we next studied interaction of the C-terminally truncated IM30 proteins with model membranes using Laurdan fluorescence spectroscopy (Figure 4). The fluorescence properties of Laurdan, a dye that integrates into lipid bilayers, strongly depends on the lipid order. When IM30 binds to Laurdan-containing membranes, the Laurdan fluorescence emission spectrum is altered, allowing evaluation of membrane binding affinities (Heidrich et al., 2016) (for details, see Material and Methods). By subtracting the GP value of a pure bilayer system from measurements of a lipid bilayer plus IM30, we obtained a ΔGP value that is proportional to the amount of Laurdan which experiences an altered environment, reflecting the amount of bound protein.

To assess whether the lipid binding propensity was altered when C-terminal IM30 helices were deleted, we determined changes in the Laurdan GP value after addition of equal amounts of proteins to liposomes. The non-physiological, neutral lipid DOPC was always used as a lipid background, and 20% of the respective TM lipids were individually mixed with DOPC to determine the lipid dependence of the IM30-membrane interaction. As can be seen in Figure 4, all truncated IM30 proteins bound specifically to membranes containing negatively charged lipids (PG and SQDG), as observed previously with the full-length protein (Hennig et al., 2015; Heidrich et al., 2017), and none of the truncations appeared to considerably reduce membrane binding.

**FIGURE 5:**
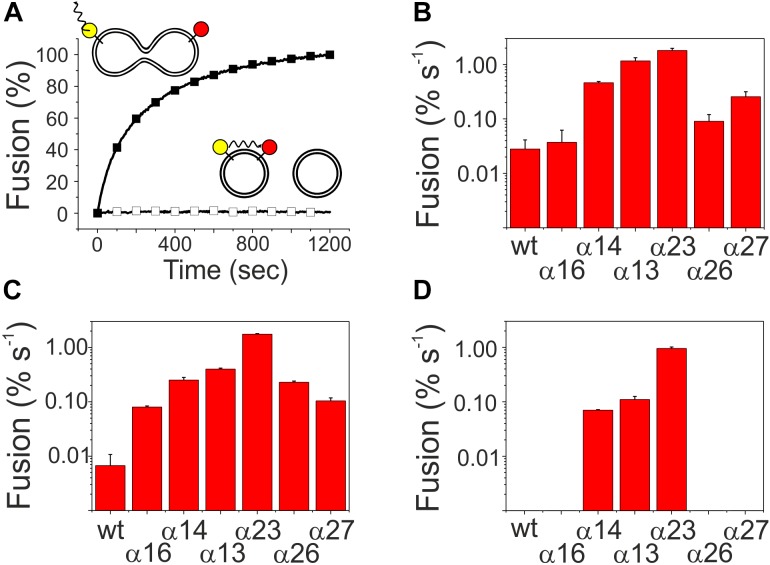
Membrane fusion activity of truncated IM30 proteins. MGDG/DOPG liposomes, containing a FRET donor (NBD, yellow) and a FRET acceptor dye (Rhodamine, red), were mixed with non-labeled liposomes. **(A)** The donor fluorescence emission, which increases upon fusion [here shown for the wt (black squares)], was monitored over time after addition of 2.5 μM protein and 7.5 mM Mg^2+^. In presence of solely Mg^2+^ no fusion is observed (black empty squares). **(B–D)** The fusion efficiency in the initial fusion phases was calculated by linear regression. Error bars represent the standard deviation of three independent replicates. The analyzed protein concentrations were 2.5 μM (B), 250 nM, **(C),** and 25 nM **(D)**.

Upon membrane binding, IM30 is able to trigger fusion of TM-mimicking membranes in presence of Mg^2+^ (Hennig et al., 2015). However, which helical domains of the protein are crucial for this fusogenic activity, is completely enigmatic yet. Thus, fusion of two liposomes was next analyzed using an established FRET assay, where LissRhod/NBD labeled liposomes were mixed with unlabeled liposomes. Upon IM30-triggered membrane fusion, the relative distance of the two fluorescently labeled lipids increases, resulting in an increased donor fluorescence (Hennig et al., 2015). A typical fusion reaction catalyzed by IM30 wt is shown in Figure 5A together with a negative control. As can be seen in Figure 5B, all C-terminally truncated proteins were able to mediate liposome fusion, with fusion activities like the wt-protein (α1-6) or even higher when fusion rates were determined using an IM30 concentration of 2.5 μM, a concentration established in a previous work (Hennig et al., 2015). To be able to better evaluate the determined fusion rates, we next performed fusion experiments using lowered IM30 concentrations (250 and 25 nM, Figure 5C,D). Even at an IM30 concentration as low as 25 nM, the proteins α1-3 and α1-4 displayed fusion rates higher than the wt, and thus, helices 4–7, and especially helix 5/6, appear to inhibit the IM30-inherent membrane fusion activity.

### Helix 1 Prevents Rod Formation and Inhibits Membrane Fusion

**FIGURE 6:**
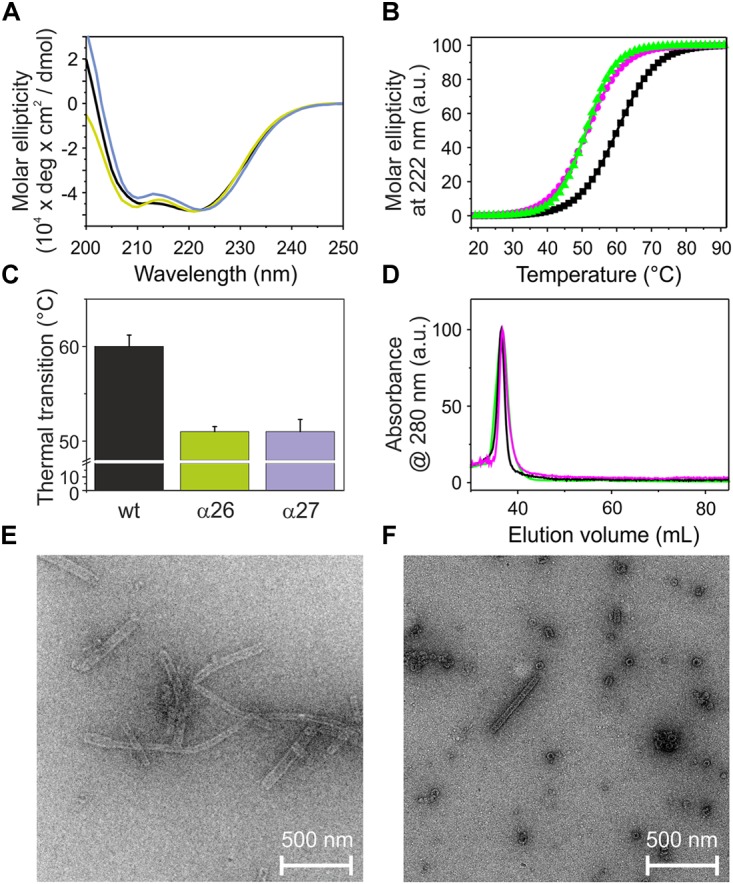
Structure and stability of N-terminally truncated IM30. **(A)** CD spectra of wt (black), α26 (green), and α27 (magenta) IM30. **(B)** The CD signal at 222 nm was followed at increasing temperatures, and the curves (melting curves) were fitted using a Boltzmann function (*R*^2^ > 0.95) yielding the transition point (i.e., the melting point) **(C)**. Error bars represent SD of three independent measurements. **(D)** Size exclusion profiles of N-terminally truncated IM30 versions. All three here analyzed proteins mainly eluted in the void volume as high molecular mass oligomers. **(E, F)** Negatively stained transmission electron micrographs of α26 **(E)** and α27 **(F)** proteins.

The analyses of the C-terminally truncated proteins have indicated an important role of helix 5/6 in ring formation and membrane fusion. To further analyze the role of the C-terminal helices in the context of helix 1, we next purified and analyzed the proteins α2-6 and α2-7 (Figure 1B,C). We refrained from generating and analyzing smaller constructs, as helix 5/6 has been identified to be key for the structure and the fusogenicity of IM30 (compare above). While the secondary structure of the two N-terminally truncated proteins is largely α-helical (Figure 6A), removal of helix 1 decreased the melting temperature of the analyzed fragments by about 9°C (Figure 6B,C), and thus the thermodynamic stability of the proteins was significantly decreased compared to the respective constructs that carry helix 1 (Figure 2).

Both analyzed proteins still form high molecular mass oligomers, as indicated in the SEC profile (Figure 6D), and TEM analyses have shown that deletion of helix 1 does not abolish formation of the IM30-specific ring structures (Figure 6E,F). However, while the IM30 wt protein only rarely forms small rods (Figure 3B; Fuhrmann et al., 2009a), extended formation of such rod structures was observed when helix 1 was absent, as observed before for the IM30 α1-6 protein (Figure 3C; Hennig et al., 2017). Consequently, the protein lacking both, helices 1 and 7 (i.e., the protein IM30 α2-6) forms rather extended rods (Figure 6E). These observations suggest that IM30 ring formation requires helices 2-6, and formation of extended rod structures via ring stacking is prevented by the two terminal helices 1 and 7. Despite these structural differences, membrane interaction of the proteins missing helix 1 is not significantly altered (Figure 4), whereas the membrane fusion rates are increased compared to the wt protein (Figure 5). Together, these results suggest that helix 1 stabilizes the IM30 secondary structure, prevents formation of extended IM30 rods and inhibits the IM30 inherent fusion activity.

### Helices 2 and 3 Are the Structural and Fusogenic Core of IM30

**FIGURE 7:**
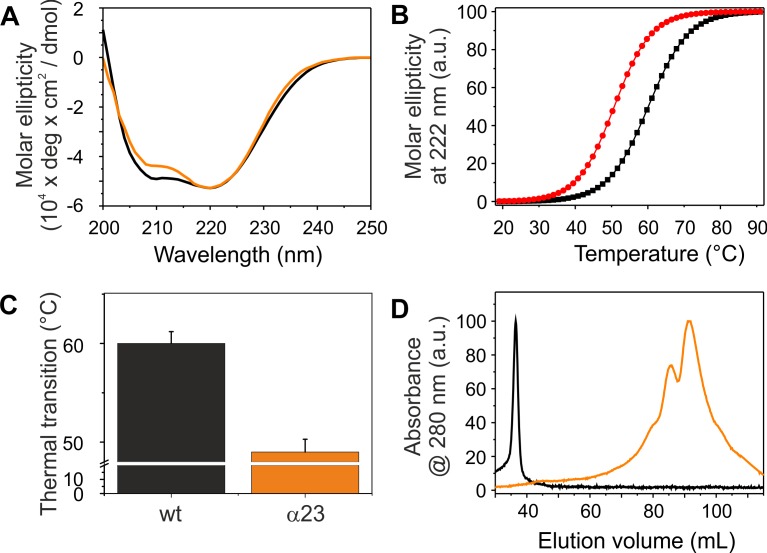
Structure and stability of IM30 α23. **(A)** CD spectra of wt (black) and α23 (orange) IM30. **(B)** The CD signal at 222 nm was followed at increasing temperatures, and the data (melting curves) were fitted using a Boltzmann function (*R*^2^ > 0.95) yielding the transition point (i.e., the melting point). **(C)** Error bars represent SD of three independent measurements. **(D)** Size exclusion profiles of wt IM30 (black) and the α23 coiled-coil (orange).

The coiled-coil formed by helices 2 and 3 represents the structural core of IM30 monomers (Figure 1A; Osadnik et al., 2015; Saur et al., 2017). The isolated helix 2/3 fragment is mainly α-helical (Figure 7A) but has a melting temperature (49°C) that is substantially lowered compared to the full-length protein (Figure 7B,C). Surprisingly, the α2-3 fragment shows two peaks in the SEC analysis with apparent molecular masses of 13.1 and 19.3 kDa (Figure 7D).While the α2-3 monomer has a calculated molecular mass of 16.8 kDa, it is likely that the peaks correspond to a monomer and a dimer (as further discussed below). Thus, the IM30 core appears to have already an intrinsic propensity to form dimeric structures. While the α2-3 fragment interacts with negatively charged membrane surfaces (at least) as stable as the full-length protein (Figure 4), the fusion rates determined with the α2-3 fragment were considerably higher than the rates determined with the wt protein or with any of the remaining truncated proteins (Figure 5). At the highest protein concentration (Figure 5B), the fusion rate was more than 60 times higher than the wt fusion activity, while it was even almost 200,000 times higher at the lowest protein concentration (Figure 5D; note the logarithmic scale in Figure 5). Thus, α2-3 might represent the fusogenic core of IM30 proteins and flanking helices are involved in dynamic shielding of this fusogenic core.

## Discussion

### Helix 2/3 Plus Helix 1 Stabilize IM30 Dimers

**FIGURE 8:**
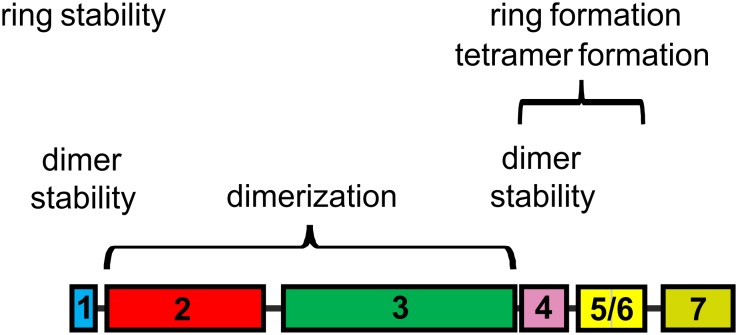
IM30 helical regions involved in structuring IM30 oligomers. Based on the present study, the central coiled-coil α23 is crucial for IM30 dimerization, whereas helices 1 and 4 are involved in stabilizing the dimer. Helices 4 and 5/6 are crucial for IM30 tetramerization and ring formation, and IM30 rings are stabilized via interactions involving helix 1. The terminal helices 1 and 7 prevent ring-ring contacts and formation of extended ring stacks. The individual helices are colored as in Figure 1.

IM30 has recently been shown to form various high molecular mass ring structures, likely from tetrameric building blocks (Heidrich et al., 2016; Saur et al., 2017). However, such high molecular mass structures are only formed by IM30 constructs that contain at least helices 2-6. Consequently, helices 1 and 7 do not appear to be directly involved in ring formation (Figure 3). Removal of additional C-terminal helices, i.e., helices 5/6 and helix 4, abolished ring formation. However, two new oligomeric states were identified when these proteins were analyzed. The coiled-coil core region formed by helices 2 and 3 appears to exist in two different oligomeric states, as indicated by the two elution peaks (Figure 7D). Noteworthy, the apparent molecular masses determined for this protein did not exactly correlate with the masses calculated for monomers and/or dimers (Table 2). For the α2-3 core structure, oligomeric states of 0.8 and 1.0 were calculated when ideal separation was assumed. However, molecular masses observed in SEC analyses often differ from calculated ones, in particular when the protein is not compact. In case of the coiled-coil structure of α2-3, a rather elongated form has to be assumed (Figure 1A), which likely explains the deviations. Furthermore, non-ideal adsorption of the proteins to the matrix may alter the retention volume and thus the determined apparent molecular masses. Therefore, the two peaks identified for the α2-3 protein likely represent a monomeric and a dimeric protein, and thus α2-3 has an intrinsic propensity to dimerize. In contrast, α1-3 and α1-4 appear to exclusively form stable dimers (Figure 3A) and the presence of helix 1 increases the thermodynamic stability of the protein (Figure 2, 7), most likely due to formation of extra inter-monomer contacts. Noteworthy, we cannot completely rule out that the two main maxima observed in the α23 SEC elution profile represent different conformations of a α23 monomer. Yet, the finding that α1-3 and α1-4 are purely dimeric and that helix 1 is not needed for formation of higher ordered structures (as outlined above) clearly supports the assumption that the α23 coiled-coil region alone has an intrinsic propensity to dimerize (Figure 8).

Based on a recent structural analysis of IM30 wt rings, it was assumed that the rings are built from tetrameric building blocks (Saur et al., 2017). Yet, it was already suggested that these tetramers represent dimers of dimers (Heidrich et al., 2016). Based on the current observations, dimerization is driven by interaction of the α2/3 coiled-coil region and IM30 dimers are stabilized via additional interactions mainly involving helix 1.

### Helices 4–6 Are Involved in Formation of IM30 Tetramers and Rings

Recently, it has been shown that individual IM30 rings assemble from tetrameric building blocks (Heidrich et al., 2016; Saur et al., 2017). Yet, in the present analyses solely regions crucial for IM30 dimerization but not for tetramer formation were identified, and all fragments that were able to form oligomers larger than dimers did form ring structures. Thus, for these truncated proteins, tetramerization inevitably resulted in ring formation, at least at the concentrations studied so far. Clearly, helix 5/6 is crucial for formation of high molecular mass oligomers, and the fragments α2-6 and α1-6 already form ring structures. This implies that dimerization of IM30 dimers and subsequent formation of oligomeric rings crucially involves helix 5/6. In a previous work, a sequence has been identified in helix 4 that is key for formation of ring structures from stable IM30 tetramers (Heidrich et al., 2016). Thus, ring formation from tetramers is likely driven via interactions of (at least) helix 4, albeit tetramer formation requires dimerization of preformed dimers, which is driven by helix 5/6 and/or interactions of helix 4 with helix 5/6 (Figure 8).

However, what role do the two remaining helices 1 and 7, i.e., the terminal helices play? The helical region 1 clearly is not directly involved in formation of IM30 rings, at least in case of the *Synechocystis* IM30 protein (Figure 3, 4). However, the oligomeric state of truncated IM30 proteins has recently been analyzed using the IM30 proteins of *Arabidopsis thaliana* and *Chlamydomonas reinhardtii* (Otters et al., 2013; Gao et al., 2015). In *Arabidopsis*, helix 1 appears to be essential for formation of high molecular weight complexes (Otters et al., 2013), whereas this was not observed as for the *Chlamydomonas* protein, and the N-terminally truncated *Chlamydomonas* protein still formed high molecular mass complexes (Gao et al., 2015). In the present study, the *Synechocystis* IM30 α2-7 and α2-6 proteins were observed to form stable ring structures. Thus, based on the results obtained with the *Arabidopsis* protein and the here presented data, helix 1 does not appear to be essential for ring formation. However, the thermal stability of α2-7 clearly was severely decreased when compared to the wt IM30 protein (Figure 6). Thus, while not needed for ring formation, helix 1 likely stabilizes IM30 ring structures. Possibly, the IM30 proteins of *Chlamydomonas* and *Synechocystis* have an overall increased stability which allows removal of the N-terminal helix 1 without abolishing ring formation, whereas in case of *Arabidopsis*, the IM30 ring structure is less stable and removal of helix 1 results in ring disassembly (Otters et al., 2013; Jovanovic et al., 2014a). Thus, we propose that helix 1 is not only involved in stabilization of IM30 dimers, as discussed above, but also in stabilizing IM30 rings.

Furthermore, the N-terminally truncated protein α2-7 had an enhanced propensity to form rod-like structures (Figure 6F), and thus, helix 1 appears to inhibit ring-ring interactions. The IM30 N-terminal region forms an amphiphilic helix (McDonald et al., 2017), and the homologous helix 1 of the *E. coli* PspA protein has been shown to interact with helix 2 (Jovanovic et al., 2014a; Osadnik et al., 2015). Possibly, helix 1 stabilizes IM30 dimers and rings via specific hydrophobic interactions with helix 2. Weakening this interaction results in exposure of a hydrophobic helix surface that is able to interact with membranes. In fact, the isolated amphipathic helices 1 of PspA and IM30 proteins have recently been shown to be able to interact with model membranes (McDonald et al., 2017). Deletion of helix 1 thus unmasks hydrophobic regions which could result in increased hydrophobic contacts between IM30 rings, i.e., in ring assembly and rod formation as observed in the present study.

Similar to helix 1, helix 7 appears to somehow shield hydrophobic IM30 regions. Deletion of helix 7 does not affect the thermodynamic stability of IM30 (Figure 2), in line with recent observations (Hennig et al., 2017), but appears to increase the propensity to form extended rod structures (Figure 3C). Furthermore, deletion of helix 7 results in increased fusion rates, most likely due to augmented exposure of hydrophobic surface regions or removal of steric hindrance. Thus, as suggested for helix 1, helix 7 is also involved in shielding individual IM30 rings from unspecific interactions.

Together, the N- and C-terminal regions of IM30 negatively regulate ring-ring contacts and artificial formation of longer ring assemblies *in vitro* (Figure 8). This became most evident when the α2-6 protein was analyzed, which was destabilized to the same degree as α2-7 but formed the most extended rods observed in this study (Figure 6E). Noteworthy, such extended structures are not observed *in vivo* and are thus likely an *in vitro* artifact (Bryan et al., 2014; Gutu et al., 2018; Junglas and Schneider, 2018).

### The Coiled-Coiled Helices 2/3 Trigger Membrane Fusion

IM30 interacts with negatively charged membrane surfaces, destabilizes membranes and induces membrane fusion in presence of Mg^2+^ (Hennig et al., 2015). As can be seen in Figure 5, the highest fusion rates were observed when solely the coiled-coil forming helix pair α2-3 was added to liposomes, whereas the fusion activity decreased when additional IM30 helices were present. This observation suggests that the helix 2/3 coiled-coil is the fusogenic core of IM30, whereas N- and C-terminal regions shield this fusogenic core and reduce the IM30-inherent fusion activity. Amphiphilic α-helical domains are well known to bind and remodel (e.g., BAR-domain containing proteins) (Salzer et al., 2017) or disrupt biological membranes (e.g., anti-microbial peptides) (Schmidt and Wong, 2013). However, such an activity must be tightly controlled in cells. Thus, while the 2/3 helical region has a high intrinsic fusion activity, ring formation could prevent uncontrolled membrane fusion. In line with this assumption, we observed increased fusion rates when IM30 rings are destabilized (Figure 5), and helix 5/6 appears to be of special importance to reduce the fusogenic activity. Since helix 5/6 was found to drive formation of IM30 rings (compare above), ring formation appears to counteract spontaneous, uncontrolled membrane fusion.

IM30 rings are thus the inactive form of IM30, and upon binding to negatively charged membrane surfaces, the fusogenic core of IM30 rings, i.e., the central helix 2/3 coiled-coil, eventually interacts with the lipid bilayer resulting in membrane destabilization and membrane fusion. This potentially involves rearrangement and/or destabilization of the IM30 ring structure and/or ring disassembly, as indicated in recent experiments (Hennig et al., 2015; Heidrich et al., 2016). Disassembly of IM30 rings results in a high local IM30 monomer concentration, which ensures locally restricted membrane destabilization and controlled membrane fusion. Thus, IM30-catalyzed membrane fusion likely requires formation of IM30 rings and controlled unmasking of the fusogenic core formed by helices 2/3. This rearrangement might be controlled by Mg^2+^, which triggers rearrangement of the IM30 ring structure (Heidrich et al., 2018), as well as by interaction of IM30 with membrane surfaces.

While we cannot rule out that membrane fusion initiated by the isolated helix 2/3 coiled-coil differs from fusion triggered by the full-length wt protein, the mechanism proposed here is very similar to the mechanism recently proposed for membrane fusion processes mediated by the human protein Synaptotagmin-1, a ring-forming protein that requires Ca^2+^ for activity (Martens et al., 2007; Hui et al., 2009; Wang et al., 2014; Zanetti et al., 2016; Wang et al., 2017). Upon Ca^2+^ binding, the so-called Ca^2+^ – loops of Synaptotagmin-1 re-orient and insert into a membrane, likely triggering ring disassembly and membrane fusion (Wang et al., 2014; Zanetti et al., 2016).

## Author Contributions

AT and DS designed the study, analyzed data, and wrote the manuscript. AT performed the experiments.

## Conflict of Interest Statement

The authors declare that the research was conducted in the absence of any commercial or financial relationships that could be construed as a potential conflict of interest.
